# Activation of Antioxidant Defenses in Whole Saliva by Psychosocial Stress Is More Manifested in Young Women than in Young Men

**DOI:** 10.1371/journal.pone.0115048

**Published:** 2014-12-19

**Authors:** Viktoriia Tsuber, Yunus Kadamov, Lydia Tarasenko

**Affiliations:** Department of Medical, Bioorganic and Biological Chemistry, Ukrainian Medical Stomatological Academy, Poltava, Ukraine; Kermanshah University of Medical Sciences, Islamic Republic Of Iran

## Abstract

Psychosocial stress has been long known to have deleterious effects on health. Nevertheless, an exposure to moderate stressors enhances resilience and promotes health benefits. Male and female organisms differ in many aspects of health and disease. The aim of this study was to investigate antioxidant activity and oxidative damage in saliva in a psychosocial stress paradigm in men and women. Here, we show that an acute stressor of moderate strength augments antioxidant activity and decreases oxidative damage in whole saliva of young people. An examination stress caused a significant increase of catalase activity, accompanied by a decrease of levels of oxidized proteins. Levels of thiobarbituric acid-reacting substances did not increase at stress, indicating that lipid peroxidation was not activated. The stress-induced alterations were more manifested in young women compared to young men. Thus, antioxidant protective mechanisms are more activated by a moderate stressor in young women than in young men.

## Introduction

The organism is constantly challenged by external and internal demands. Rapid behavioral and physiological adjustments to the change of conditions are often necessary to maintain homeostasis. Stress reaction is essential to ensure an appropriate response and promote adaptation [1]. Turning on the machinery of stress response facilitates coping with immediate changes of the environment, thus providing survival benefits. However, excessively prolonged or frequent activation of stress response has deleterious health consequences in the long run. Adverse effects of stress are multifaceted and have been extensively documented by numerous researchers.

Nevertheless, despite the universally acknowledged harmful effects of stress a growing body of evidence suggests that activated stress response can promote health benefits under certain conditions. An exposure to stress may strengthen an organism's resilience and resistance to noxious agents. There are multiple findings that associate moderate short-term stress with enhanced immunity [2], [3]. Stress-related activation of the sympathetic adrenal medullar system is accompanied by an elevation of immune cell numbers [4] or an increase of sIgA levels in saliva [5]. It has been shown that acute stress stimulates leukocyte trafficking and cytokine gene expression at the site of antigen entry, thus strengthening skin immunity [6]. Academic examination stress was associated with increased mitogen-stimulated lymphocyte proliferation [7], [8]. It may be assumed that stimulation of immune system by acute stress may provide survival benefits through an increased readiness for traumas and wounds [9]. Thus, short-term stressors may boost resilience and improve performance in dealing with frequently occurring stressful experiences [10] and lead to growth, adaptation and beneficial learning that promote stress resistance and good health [11].

Though it has been long recognized that increased oxidative damage is a factor in pathophysiology of stress-induced lesions and depression [12], [13], [14], recent findings add a twist to the established picture suggesting that acutely stressful events may not always result in aggravated oxidative injury. A study by Aschbacher et al. (2013) demonstrated that enhancement or weakening of organism's resilience to oxidative damage depends on the individual's prior exposure to chronic psychological stress [15]. Furthermore, increased levels of perceived stress were associated with alleviation of oxidative damage, but only among women with low levels of chronic perceived stress [16]. Similarly, it has been found that an exposure to a moderate stressor boosts resilience to oxidative damage in postmenopausal women [15].

Recent advances have shown that men and women exhibit marked differences in terms of disease symptoms, prognosis, psychological and social impact [17]. Additionally, a substantial amount of data states that men and women differ in handling stressful situations [18], [19] and in manifestation and extent of adverse effects caused by stress [20], [21]. Our results show that they also differ in beneficial effects of stress response. In this study, we found that acute psychosocial stress enhances antioxidant activity and diminishes manifestations of oxidative damage in whole saliva of young people. We also report substantial differences between women and men in the stress response.

## Materials and Methods

### Subjects

103 healthy young undergraduates (45 male, 58 female, mean age  = 19.79, SEM = 0.14) volunteered to participate in the study. One woman did not return for the second saliva collection, therefore her data were excluded from the study. The subjects were all free of use of medication. The study protocol was approved by the Ethics Committee of the Ukrainian Medical Stomatological Academy, Poltava, Ukraine prior to the study. The subjects were informed about the aim and procedure of the study and each subject signed an informed consent form. Potential subjects were excluded if they suffered from an upper respiratory tract infection, allergic or inflammatory response at the moment of saliva collections. Participants filled out a self-report questionnaire on health (perceived health, use of medication, oral hygiene and dental health). Participants were instructed to abstain from eating, drinking or engaging in physical exercise for one hour prior to the experiment. 29 of the participants were light to moderate smokers. They reported smoking between 3 and 10 cigarettes per day.

### Experimental design and saliva collection

In this experiment saliva samples were provided by subjects at two time points. The first sample represents a baseline condition. It was taken during a period that was relatively stress-free for the participants, approximately two weeks prior to the stress condition. The stress condition was an important academic examination at the end of the term. The second sample was taken immediately before the start of the examination. The participants completed a self-rating questionnary on anxiety before providing saliva samples. The both stages of the experiment took place at 09:00 h in the morning to avoid influence of the circadian rhythm on the parameters under study.

Unstimulated whole saliva was collected by passive drooling for 7 minutes into preweighed test-tubes. After collection, saliva was clarified by centrifugation (3 000 rpm, 5 minutes) to eliminate buccal cells and oral microorganisms. The clear supernatant was divided into 500-µl aliquots and stored at −20°C until use. Saliva flow rate and saliva density were determined for the samples.

While for all the subjects anxiety data were calculated and salivary alpha amylase activity was measured, the thiobarbituric acid-reacting substances (TBARS) concentrations and levels of oxidatively modified proteins were assayed in whole saliva of 36 subjects (14 men, 22 women). Activity of catalase and concentration of sialic acids were measured in saliva of the other 66 subjects (31 men, 35 women). Of these, catalase activity was assayed in saliva of 52 subjects (24 men and 28 women), sialic acids were measured in saliva of 44 subjects (21 men, 23 women), because not all subjects provided enough saliva for complete analysis.

### Data analysis

All measurements were tested for normality with the Kolmogorov-Smirnov test. No transformations were necessary for any of the variables. Prior to analysis, outlying data (i.e data that exceeded their respective group means by more than two standard deviations) were winsorized to be 5% greater than the next largest value. By winsorization statistical artifacts can be avoided for small sample sizes. Student's paired-samples t-tests were computed to reveal differences between the baseline and the stress conditions. Student's independent-samples t-tests were used to analyze differences between men and women. To analyze associations between variables, Pearson correlations were computed.

All analyses were also performed restricted to those participants who did not smoke. The results were similar to those of the entire group.

For all statistical analyses, R statistical computing program was used. Data are expressed as mean ±SEM. A *p* value of 0.05 (two-tailed) or lower was considered statistically significant.

### Psychological measures

To measure the subjective significance of the examination as a stressor we used the State and Trait Anxiety Inventory (STAI) [22], [23]. It is a valid and reliable measure, with Cronbach's α ranging from 0.89 to 0.94 [24]. The STAI is one of the most commonly used scales to assess anxiety levels in students [25]. It has been validated for many countries, including Ukraine [26]. The psychometric data for the population of young people in Ukraine do not differ from those in Western Europe [26]. The STAI consists of two 20-item scales, representing sets of questions reflecting the trait and the state anxiety. Each item is rated on a Likert 4-point scale, where “1” means “not at all” up to “4” that means “very much”. The state anxiety scale measures immediate feelings of anxiety, while the trait anxiety scale indicates stable individual predisposition to anxiety. In this work, only the state anxiety data are used.

### Alpha amylase

Alpha amylase activity (mg/s×l) was determined using a commercially available assay kit (Felicit Diagnostics, Ukraine). The reagents in the kit contain a preparation of starch as an alpha amylase substrate. 0.02 ml of saliva were added to 0.5 ml of the buffered substrate and incubated for five minutes at 37°C. The reaction was then quenched by adding 4.5 ml of an inhibitor solution. 0.05 ml of iodine was added to react with the rest of substrate, that was not transformed by alpha amylase action. The rate of degradation of the substrate is directly proportional to the amylase activity. The activity was determined by measuring the absorbance at 640 nm against distilled water.

### Catalase

Catalase activity (mcat/l) was determined by a spectrophotometric measurement of breakdown of hydrogen peroxide in a reaction with ammonium molibdate [27]. 2 ml of 0.03% H_2_O_2_ solution were incubated with 0.1 ml of saliva for ten minutes. Then, 1 ml of 4% ammonium molibdate solution was added and the absorbance of the final sample was measured at 410 nm against a reagent blank.

### Protein concentration

Protein concentration (g/l) was measured by the biuret method [28]. 0.2 ml of saliva or of a standard protein solution was mixed with 1.8 ml of distilled water and 2 ml of 6% sodium hydroxyde solution. Then, 0.2 ml of the Benedict's reagent (17.3 g of sodium citrate, 10 g of sodium carbonate, 1.73 g of copper sulfate pehtahydrate in 100 ml of water) was added and the mixture was incubated for fifteen minutes. The absorbance was measured at 330 nm against a reagent blank. Bovine serum albumin was used as a standard.

### Oxidatively modified proteins

Amount of oxidatively modified proteins (mol^−1^cm^−1^) was measured by derivatization of carbonyl groups with dinitrophenylhydrazine (DNPH) [29]. DNPH forms Schiff bases with carbonyl groups. 1 ml of solution of 20% trichloroacetic acid and 1 ml of 0.1 *M* DNPH was added to 1 ml of saliva. The samples were incubated for 60 minutes. Then, the samples were centrifuged for thirty minutes at 3000 rpm. The precipitate was washed with 1 ml of 2 *M* HCl three times to remove the residual DNPH. Then, the precipitate was dissolved in 2 ml of 6 *M* urea. The absorbance was measured at 370 nm against 6 *M* urea.

### TBARS

The TBARS assay (mcmol/l) was used as a measure of lipid peroxidation [30]. 1 ml of solution of 17% trichloroacetic acid and 1.5 ml of distilled water was added to 1 ml of saliva. The samples were centrifuged for ten minutes at 3000 rpm. 2.0 ml of the supernatant solution was added to 1.0 ml of thiobarbituric acid. The tubes were capped and heated in a boiling water bath for ten minutes. The samples were cooled and the absorbance was measured at 532 nm against a reagent blank.

### Sialic acids

Concentration of sialic acids [31] (mg/l) was measured by the Hess method in a reaction with sulfuric acid. 0.5 ml of solution of 10% trichloroacetic acid was added to 0.5 ml of saliva to precipitate salivary proteins. The samples were placed in a boiling water bath for five minutes. After heating the tubes were cooled in an ice bath for five minutes and centrifuged for five minutes at 1500 rpm. 0.4 ml of the supernatant solution was added to 5 ml of the Hess reagent (5 ml of concentrated sulfuric acid in 95 ml of glacial acetic acid). The tubes were capped and heated in a boiling water bath for thirty minutes. The samples were cooled and the absorbance was measured at 540 nm against a reagent blank.

## Results

The means and standard errors of means (SEM) for all the parameters in the study are presented in S1 Table. All the data on which the study is based are listed in S2 Table.

### Salivary flow rate and protein concentration in saliva at psychosocial stress

Salivary flow rate is expressed as the total amount of saliva secreted in one minute (mg/min). Salivary flow rates did not change significantly from rest to stress. The measures of salivary flow rate were significantly correlated between the conditions (r(102)  = 0.674, p<0.01). No difference of flow rate between men and women was found at the both conditions.

We did not find differences in salivary total protein concentration (mg/ml) at rest and at stress. A weak but significant association of salivary protein levels between two conditions was observed (r(102)  = 0.299, p = 0.002). Men and women did not demonstrate significant differences in protein concentrations at rest. A slightly higher salivary protein concentration was found in men compared to women at stress, the difference was however only marginally significant. Correlational analysis revealed, that there were no associations of salivary flow rate or total protein concentration with any of the parameters in the study.

### Effect of stress on anxiety levels and activity of salivary alpha amylase

**Figure 1:**
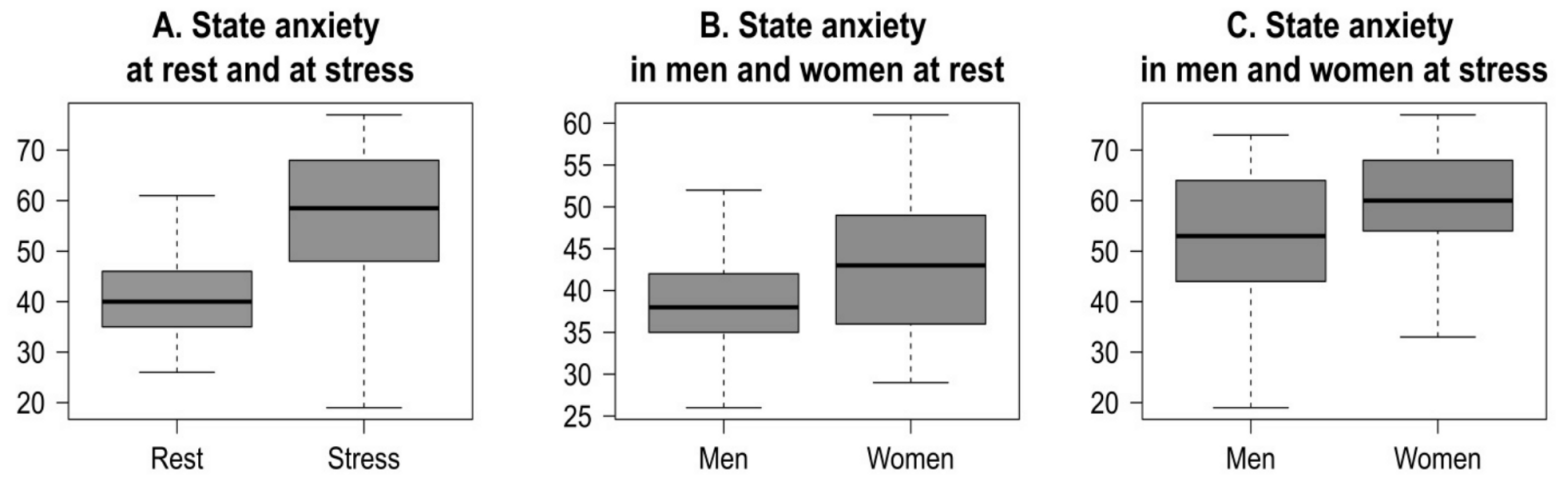
State anxiety. State anxiety of young people at rest and at psychosocial stress situation (A); state anxiety of men and women at rest (B) and at stress (C).

We examined State Anxiety (SA) scores as a measure of perceived stressfulness of the experience. Fig. 1 summarizes alterations of state anxiety at psychosocial stress experience.

Academic examinations are potent stressors that cause an increase in anxiety. A stress-induced anxiety rise compared to an exam-free period proves that the examination was the main stressor experienced by the subjects at the time of data collection. Participants rated the academic examination as significantly more stressful than the control condition, with a mean 38% increase in state anxiety (Fig. 1A). Baseline SA scores were significantly correlated with SA scores at stress (r (102)  = 0.393, p<0.01). In all measurements anxiety was higher in women than in men. There were significant differences in resting levels of state anxiety between women and men (Fig. 1B). Similarly, at stress women also demonstrated significantly higher SA levels compared to men (Fig. 1C).

**Figure 2:**
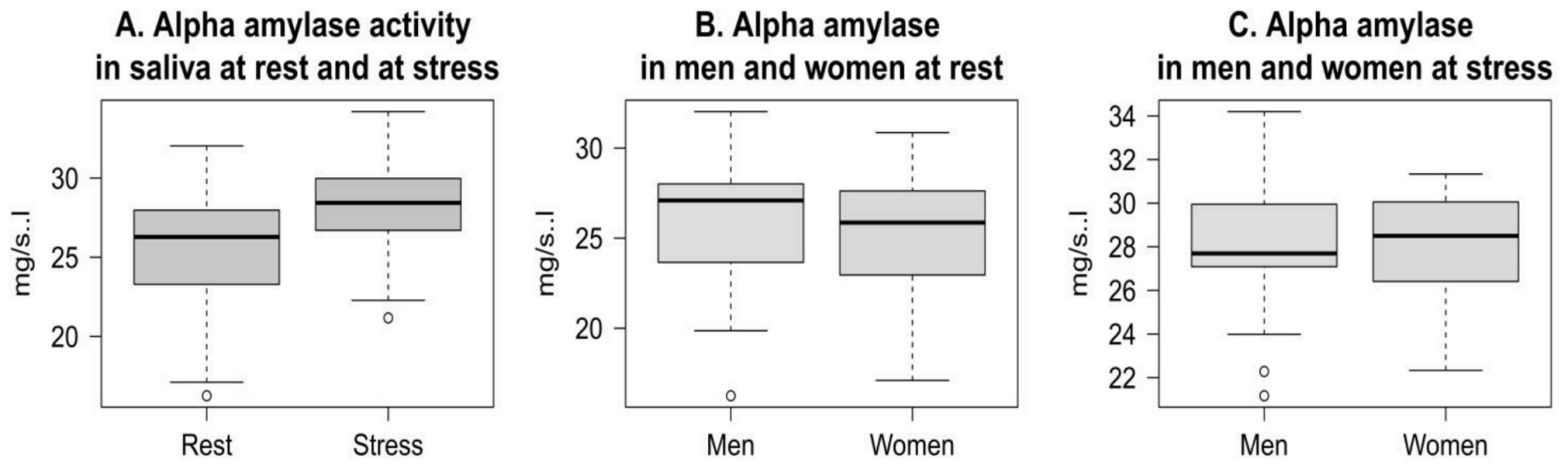
Activity of alpha amylase. Activity of alpha amylase in whole saliva of young people at rest and at psychosocial stress situation (A); activity of alpha amylase in saliva of men and women at rest (B) and at stress (C).

Salivary alpha amylase activity is a highly sensitive marker of the sympathetic adrenal medullar system (SAM) activation in the stress response. Fig. 2 shows effect of psychosocial stress on alpha amylase activity in whole saliva.

Alpha-amylase activity showed a highly significant increase in response to psychosocial stress compared to baseline (Fig. 2A). Alpha-amylase levels did not differ significantly between men and women at rest (Fig. 2B) as well as at stress (Fig. 2C). Correlation analysis showed no association of data of state anxiety with alpha amylase measurements at both conditions. The correlations were far from statistical significance: for baseline r(91)  = 0.080, p>0.05, for the stress condition r(91)  = 0.190, p>0.05. Also, no significant relationship was observed between alpha-amylase levels at rest and at stress (r(91)  = −0.048, p>0.05).

### Effect of stress on prooxidant – antioxidant balance

**Figure 3:**
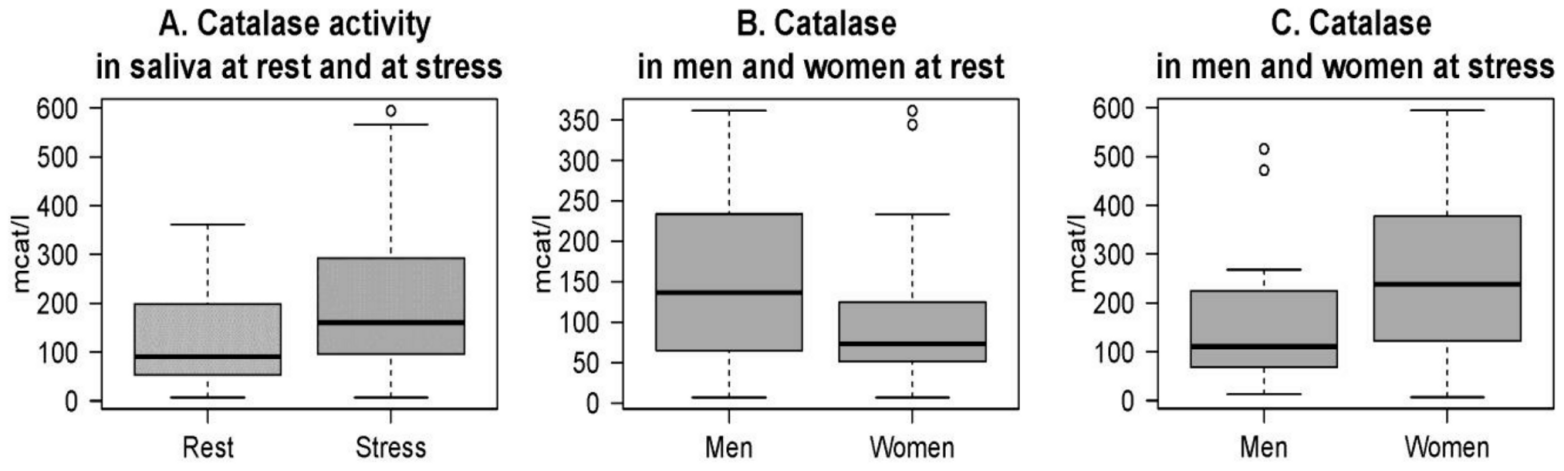
Activity of catalase. Activity of catalase in whole saliva of young people at rest and at psychosocial stress situation (A); activity of catalase in saliva of men and women at rest (B) and at stress (C).

We tested effects of stress on activity of antioxidant enzyme catalase. We found a highly significant increase of catalase in saliva at stress compared to baseline (Fig. 3A).

Variance in catalase levels is relatively high in both men and women. No association was found between catalase levels at stress and at rest (r(52)  = 0.058, p>0.05). However, an analysis of sex differences in catalase activity revealed that the stress-induced rise was due to much stronger stress response of catalase in women. At baseline no significant difference was observed in catalase levels between males and females (Fig. 3B). Psychosocial stress induced a mean 126% increase of catalase activity in saliva of women compared to baseline, while a much smaller rise was observed in men (Fig. 3C). The difference between men and women at stress is highly significant.

**Figure 4:**
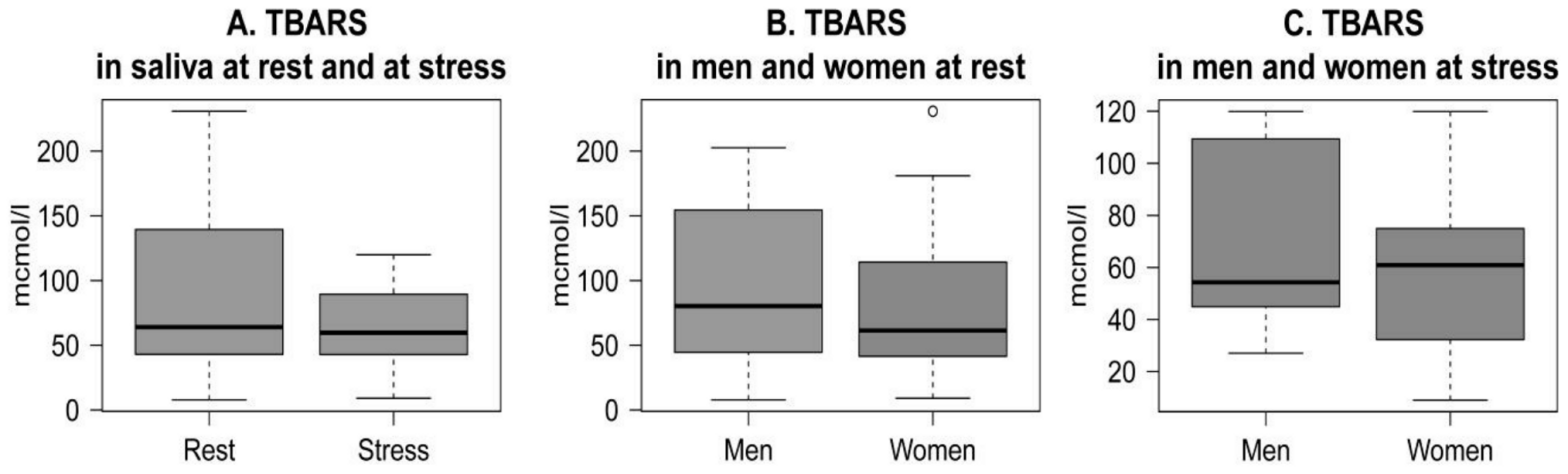
Levels of TBARS. TBARS in whole saliva of young people at rest and at psychosocial stress situation (A); TBARS in saliva of men and women at rest (B) and at stress (C).

Concentrations of thiobarbituric acid-reacting substances (TBARS) did not show alterations from baseline to stress condition (Fig. 4A).

High variance of TBARS levels was observed at both conditions. TBARS levels were not associated between baseline and stress (r(36)  = 0.047, p>0.05). No significant difference was observed in men and women at rest (Fig. 4B) or at stress (Fig. 4C).

**Figure 5:**
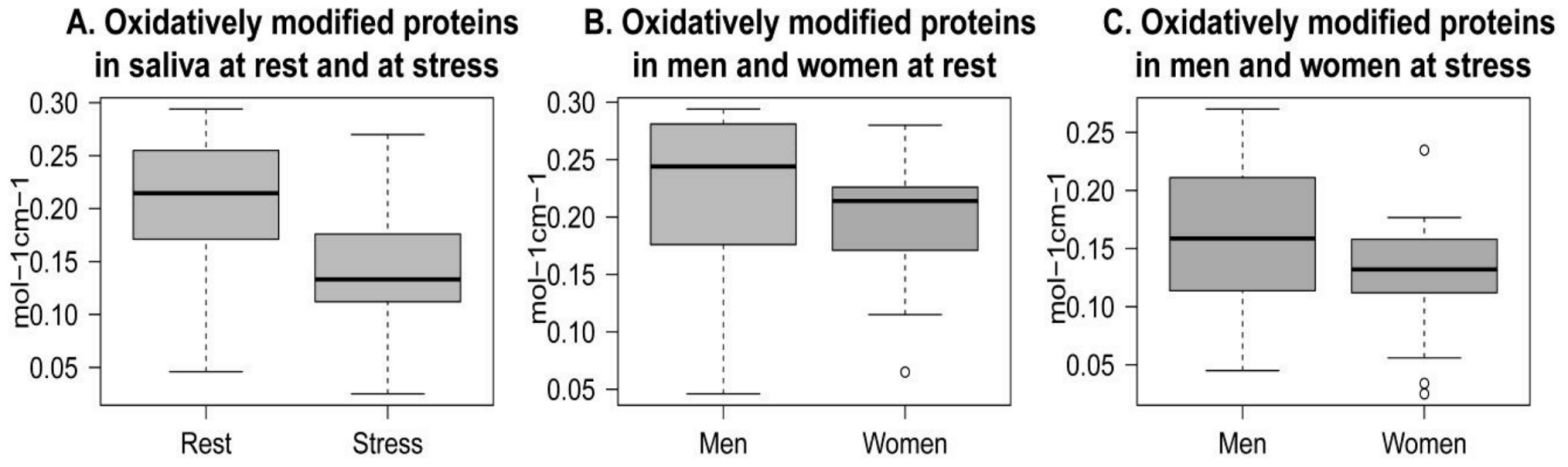
Levels of oxidatively modified proteins. Oxidatively modified proteins in whole saliva of young people at rest and at psychosocial stress situation (A); oxidatively modified proteins in saliva of men and women at rest (B) and at stress (C).

We analyzed how psychosocial stress effects concentration of oxidatively modified proteins in whole saliva. In Fig. 5 we show stress-induced changes of levels of oxidatively modified proteins.

A statistically significant decrease of oxidized proteins was observed at stress compared to rest condition (Fig. 5A). A marginally significant positive correlation was found between levels of oxidized proteins at the two conditions (r(34)  = 0.336, p = 0.052). No difference was found between men and women at rest (Fig. 5B). However, psychosocial stress caused a marginally significant greater decrease of oxidized proteins in women compared to men (Fig. 5C).

**Figure 6:**
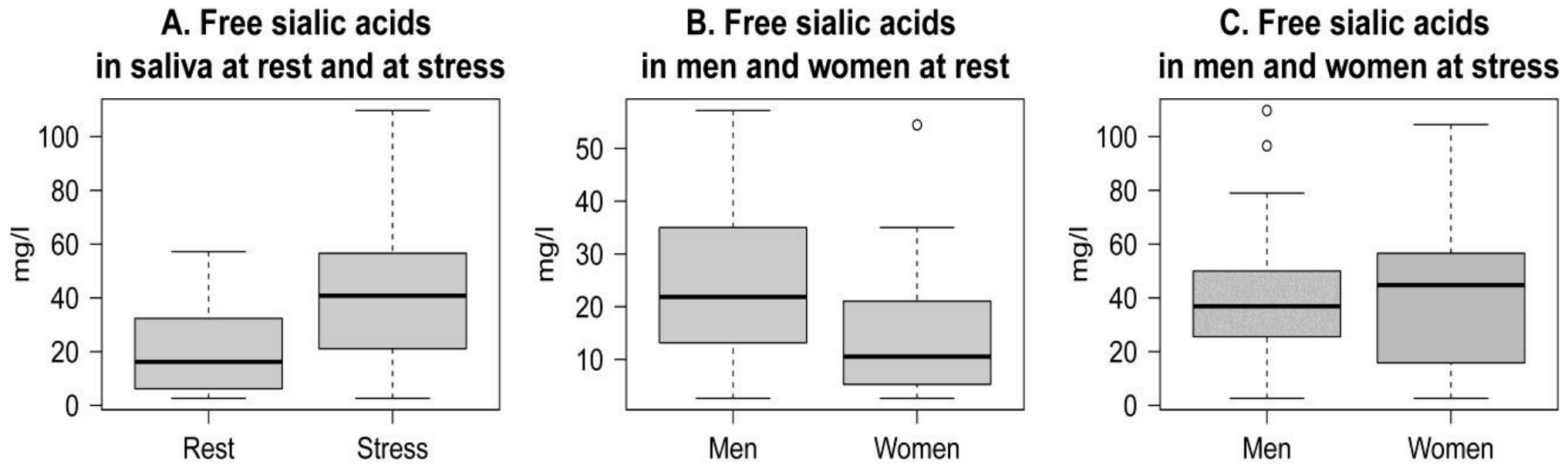
Levels of free sialic acids. Free sialic acids in whole saliva of young people at rest and at psychosocial stress situation (A); free sialic acids in saliva of men and women at rest (B) and at stress (C).

We found a more than twofold highly significant increase in concentration of sialic acids in saliva in response to stress compared to the rest condition (Fig. 6A).

No association was observed between baseline and stress levels of sialic acids (r(44)  = 0.169, p>0.05). Resting levels of sialic acids were significantly lower in women than in men (Fig. 6B). The levels of sialic acids increased in both sexes at stress and did not differ significantly in women and in men (Fig. 6C). Thus, we found a greater rise of free sialic acids in saliva of women compared to men.

## Discussion

Our research focuses on how antioxidant activity and oxidative damage in saliva is affected in a psychosocial stress paradigm in men and women. Numerous studies report differences between males and females in response to stress. Men and women handle stressful situations differently [18] and exhibit differences in cortisol responses [20], [32], [33], [34]. Women differ from men in emotionally driven memory performance [35], [36] or feelings of sadness and anxiety following stress [37]. The differences in stress reactivity may have implications for certain aspects of physiological reactions and overall health.

In the present study, the highly stressful situation of academic examination induced a significant rise of state anxiety, with mean increase of 38%. Salivary alpha-amylase levels were measured to verify activation of sympathetically mediated responses to psychosocial stress. The principal function of salivary alpha-amylase is initial digestion of dietary homopolysaccharides in the oral cavity. The enzyme is synthesized and secreted by acinar cells of the major salivary glands, the process that is mainly regulated by the autonomic nervous system through sympathetic and parasympathetic innervation [38] and modulated by beta-adrenergic receptors. Increased levels of salivary alpha-amylase have been proven to be a marker of the SAM activation as part of the stress response [39], [40]. Salivary alpha-amylase is a highly valid parameter reflecting alterations induced by psychosocial stressors [38] that is more sensitive to psychological stress than blood pressure or heart rate [40]. Stress-induced increases of salivary alpha-amylase activity are independent of flow rate and sampling method [41]. We found that acute psychosocial stress induced a significant increase of salivary alpha-amylase activity in the stress paradigm. Combined, the rise of state anxiety and the concomitant increase of salivary alpha-amylase activity indicates the potency of the stressor experienced by the participants and emphasizes the scope of the stress-induced mobilization of antioxidant activity as a means of stress protection.

The levels of state anxiety at stress were significantly higher in women than in men at stress and at rest. However, we did not find any significant difference of alpha-amylase levels at baseline and at stress between men and women. Furthermore, no sex differences were observed in the absolute and relative increases of alpha-amylase activity in response to stress that is consistent with studies of impact of sex on basal activity of salivary alpha-amylase [42], [43] and on acute salivary alpha-amylase stress responses [44], [45]. The absence of sex-specific differences of stress-induced increases in salivary alpha-amylase activity might indicate involvement of pathways other than SAM activation regulating different antioxidant response in men and women.

Oxidative alterations are important factors in virtually all processes in the organism. It is known that under normal conditions 1–3% of all electrons produced by the mitochondrial electron transport chain are diverted to generation of superoxide, that can further interact with other molecules to produce other reactive species [46]. ROS are thus a byproduct of aerobic metabolism [47] that can damage components of the cell because of their high chemical reactivity. Evidence from a growing body of literature suggests importance of excessive oxidative stress in disease incidence, severity, morbidity and mortality [48], [49], [50], [51]. Psychosocial stress is a potent contributor to oxidative damage [52], possibly due to production of free radicals in autooxidation of catecholamines [53]. However, recent research suggests, that psychosocial stress can sometimes lead to augmented resilience to oxidative damage [15], [16]. Our findings indicate, that acute psychosocial stress can result in robust activation of antioxidant defenses and a decrease of oxidative damage. In the present work, examination stress resulted in a significant increase of catalase activity and a decrease of levels of oxidized proteins in whole saliva of young people. We did not find change of TBARS levels between rest and stress conditions indicating that the stressful experience failed to intensify lipid peroxidation.

Men and women differ in many aspects of health as well as exhibit marked differences in disease symptoms, prognosis, psychological and social impact [17]. Men are known to be more vulnerable to a variety of diseases, for instance, atherosclerotic cardiovascular diseases [54], [55]. Oxidative stress is one of the main factors involved in pathophysiology of the diseases [56]. The difference is primarily attributed to greater antioxidant capacity of the female organism. Compared to male mitochondria, those of females have higher levels of superoxide dismutase, glutathione peroxidase and reduced glutathione due to estrogen-mediated expression of the antioxidant enzymes [57]. Thus, female mitochondria are better protected against adverse effects of reactive oxygen species [58]. A number of studies found differences of baseline oxidative status/oxidative stress in males and females. Ide et al. (2002) reported significantly higher levels of malonic dialdehyde and higher excretion of a marker of lipid peroxidation urinary 8-isoprostaglandin F_2α_ in healthy young men compared to age-matched women [59].

Catalase is one of the three most important antioxidant enzymes, the other two being superoxide dismutase (SOD) and glutathione peroxidase. It mediates detoxification of endogenous hydrogen peroxide. Augmented catalase expression may possibly correlate with life span [60], [61] and improves the ability of mitochondria to synthesize ATP [62]. In the present study, we observed a highly significant rise of catalase activity in whole saliva of young people upon exposure to an acute psychosocial stressor. We did not find baseline difference between men and women in catalase levels in saliva. Likewise, no significant sex-specific differences in levels of total SOD and catalase in blood plasma and in erythrocytes were found in a study of 860 men and 922 women [63]. Consistently, Ide et al. reported no difference in activity of the enzymes in blood plasma of men and women [59]. Thus, baseline antioxidant activity in women does not differ from that in men. However, we demonstrated that stress-induced increase of catalase activity in saliva is much greater in women than in men and is related to reduced oxidative damage. The underlying molecular mechanisms that augment catalase activity in women are unknown. Similarly, there is no unified concept of possible sources of catalase in saliva. A study by Nickerson et al. in 1957 presumed that catalase origin in stimulated saliva was bacterial [64], while an earlier report by Eggers-Lura claimed, that catalase was present in the parotid gland secretion [65]. To the best of our knowledge, no other findings have provided a conclusive proof of any of the notions by the present moment. However, several studies investigated catalase activity in saliva. A significant increase in activity of catalase, superoxide dismutase and peroxidase was found in saliva of young women after one month course of aerobic training [66], while decreased activity of the three antioxidant enzymes was reported in vegetarians [67]. We believe that salivary catalase in women is a part of intrinsic antioxidant defense. An argument in favor of the hypothesis is the fact that all subjects of our study reported good oral health and oral hygiene, and it is therefore highly unlikely that impaired hygienic habits at stressful circumstances could bring about increased catalase activity from bacteria in women but not in men.

One of common measures of oxidative stress is the amount of the end products of lipid peroxidation [68]. The TBARS assay collectively measures lipid peroxidation products, of which malondialdehyde is the most abundant constituent [69]. Levels of TBARS in biological fluids increase in a variety of diseases associated with oxidative stress [70]. In the present study, we did not find an increase of TBARS levels in whole saliva in response to an acute psychosocial stressor that might indicate resilience to lipid peroxidation in saliva of young people at stress, possibly because of the enhanced activity of catalase. No difference in TBARS levels was observed between men and women at rest as well as at stress.

The side chains of all amino acid residues of proteins are susceptible to oxidation by ROS [71]. Excessive oxidative stress results in carbonylation of proteins that is an irreversible oxidative modification often leading to decrease or loss of protein function [72], [73]. The concentration of carbonyl groups is a good measure of ROS-mediated protein oxidation [46]. Unexpectedly, in the present study we found that levels of oxidatively modified proteins responded to stress with a substantial decrease that was larger in women than in men. The decrease might be explained by augmented catalase activity and is consistent with the observation that stress caused a greater rise in catalase in women compared to men.

In the present study we investigated how psychosocial stress effects levels of free sialic acids in whole saliva. Sialic acids are believed to be important in antioxidant defense [74], [75]. They occupy the terminal position of many glycoproteins, e.g. mucins and have important roles in their functioning. Mucins have been found to be hydroxyl radical scavengers, with sialic acid moieties essential for the role [74]. However, excessive quantities of hydroxyl radicals cause depolimerization of native mucin [76], while Eguchi et al. showed that the glycosidic linkage of sialic acid is a potential target for superoxide and other related ROS that therefore cause liberation of free sialic acids [77]. Iijima et al. showed that free N-acetylneuraminic acid scavenges hydrogen peroxide under physiological conditions and is a potent defense molecule against oxidative stress [75]. We have found a significant stress-induced increase of free sialic acids in whole saliva of young people at stress. Interestingly, baseline levels of free sialic acids were significantly lower in women than in men. An exposure to a psychosocial stressor caused a sharper increase of sialic acids in saliva of women and resulted in similar concentration of sialic acids in both sexes. The mechanism and the role of the observed stress-induced rise of free sialic acids in saliva is unclear. The rise might be attributed to the intensified damage of mucins at stress. Alternatively, the phenomenon might indicate an additional protective mechanism against oxidative lesions.

We tested effects of psychosocial stress on salivary flow rate and total protein concentration to eliminate the possibility that they are confounding factors in the research. Up to now research of effects of stress on salivary flow rate or on total protein concentration in saliva has produced contradictory results. A decreased flow rate was found in an academic stress paradigm [78]. By contrast, an increase in flow rate was reported in response to acute stressful mental task [79], watching a stressful video [80] or the Trier Social Stress Test paradigm [41]. No change in salivary flow rate was observed by Takai et al. (2004) [81] in watching stressful and soothing videos or by Naumova et al. (2012) [82] in response to public speaking. Likewise, the currently available literature is inconclusive regarding levels of total protein in saliva at stress. Several studies report stress-induced increases of total protein concentration in saliva [82], [83], [84]. Conversely, no differences in total salivary protein were observed between stressed and non-stressed subjects [85], [86]. In the present study, no significant differences of salivary flow rate or total protein concentration were found for both conditions. Also, no associations were found between the basic salivary measurements and the markers of prooxidant-antioxidant balance. Therefore, we can assume that the observed changes of prooxidant-antioxidant markers in response to stress were not due to unchanged levels of catalase, oxidatively modified proteins, malonic dialdehyde or sialic acids in an altered volume of saliva or a different amount of protein.

The study has a number of limitations. Extrapolation of our findings may be limited to young healthy people. Additionally, we were only able to assess prooxidant-antioxidant changes at the onset of stress, while different patterns of response may emerge over longer time periods.

## Conclusions

Taken together, the present findings suggest that an acute psychosocial stressor of moderate strength might produce a beneficial effect in young people through intensified antioxidant activity and alleviated oxidative damage in whole saliva. We further demonstrate that the observed activation of antioxidant defenses is more potent in young women than in young men. The specific mechanisms by which antioxidant protection is markedly upregulated in women remain to be elucidated and might have implications in treatment of diseases associated with increased oxidative stress. Additionally, our data indicate the necessity of further research needed to conclusively establish the source, role and regulation of the important antioxidant enzyme catalase in saliva.

## Supporting Information

S1 Table **Means and standard errors of means for the tested parameters.**
(PDF)

S2 Table **Set of data.**
(XLSX)
