# Supplementary material for: Activation of Antioxidant Defenses in Whole Saliva by Psychosocial Stress Is More Manifested in Young Women than in Young Men
Source: PLoS One. 2014 Dec 19;9(12):e115048. doi: 10.1371/journal.pone.0115048 (PMC4272280; doi:10.1371/journal.pone.0115048)
Supplement: S1 Table — Means and standard errors of means for the tested parameters. (PDF) [file pone.0115048.s001.pdf]

Table S1. Means and standard errors of means for the tested parameters

	**Rest all subjects** mean SEM	**Stress all subjects** mean SEM	t-test df	p (two-tailed)	**Rest**				**Stress**			
					**Women** mean SEM N	**Men** mean SEM N	t-test df	p(two-tailed)	**Women** mean SEM	**Men** mean SEM	t-test df	p(two-tailed)
Salivary flow rate	501.48<br>26.12	505.19<br>25.70	0.18<br>101	0.860	474.56<br>34.22<br>57	535.58<br>40.20<br>45	1.16<br>100	0.248	489.63<br>31.75	524.89<br>42.39	0.68<br>100	0.499
Salivary total protein	2.51<br>0.10	2.56<br>0.10	0.41<br>101	0.684	2.38<br>0.13<br>57	2.67<br>0.14<br>45	1.51<br>100	0.136	2.38<br>0.15	2.78<br>0.14	1.95<br>100	0.054
State anxiety	40.99<br>0.79	56.57<br>1.28	12.85<br>101	p<0.001***	43.02<br>1.13<br>57	38.42<br>0.95<br>45	3.01<br>100	0.003**	59.77<br>1.50	52.51<br>2.07	2.91<br>100	0.004**
Salivary alpha amylase	25.45<br>0.36	27.99<br>0.26	5.97<br>90	p<0.001**	25.09<br>0.47<br>52	25.93<br>0.54<br>39	1.18<br>89	0.243	27.90<br>0.35	28.10<br>0.41	0.38<br>89	0.704
Catalase	130.90<br>14.59	214.10<br>22.62	3.18<br>51	0.003**	115.48<br>20.78<br>28	148.90<br>20.14<br>24	1.15<br>50	0.258	261.03<br>33.42	159.24<br>26.18	2.34<br>50	0.020*
TBARS	84.79<br>10.41	66.08<br>5.25	1.64<br>35	0.111	76.82<br>12.83<br>22	97.32<br>17.67<br>14	0.96<br>34	0.344	62.30<br>6.57	72.02<br>8.77	0.90<br>34	0.375
Oxidatively modified proteins	0.21<br>0.01	0.14<br>0.01	5.41<br>33	p<0.001***	0.20<br>0.01<br>21	0.22<br>0.02<br>13	0.74<br>32	0.463	0.13<br>0.01	0.17<br>0.02	2.03<br>32	0.050*
Sialic acids	19.36<br>2.25	43.28<br>4.29	5.32<br>43	p<0.001***	15.02<br>2.73<br>23	24.10<br>3.43<br>21	2.09<br>42	0.043*	43.58<br>6.36	42.96<br>5.85	0.07<br>42	0.943

Rest denotes the control condition and Stress denotes the psychosocial stress experience.
* p≤0.5
** p≤0.01
*** p≤0.001
